# Enhancing Skin Wound Healing in Diabetic Mice Using SIKVAV-Modified Chitosan Hydrogels

**DOI:** 10.3390/molecules29225374

**Published:** 2024-11-14

**Authors:** Xionglin Chen, Xiaoming Cao, Jie Zhang, Chen Jiang, Yitian Yu, Hui Chen

**Affiliations:** 1 Jiangxi Provincial Key Laboratory of Cell Precision Therapy, Department of Histology, Embryology and Medical Genetics, School of Basic Medical Sciences, Jiujiang University, Jiujiang 332000, China; 6140108@jju.edu.cn (J.Z.); 2 Department of Anatomy, School of Basic Medical Sciences, Jiujiang University, Jiujiang 332000, China; 6140067@jju.edu.cn (X.C.); 6140041@jju.edu.cn (H.C.); 3 Department of Oral Medicine, Grade 2021, School of Basic Medical Sciences, Jiujiang University, Jiujiang 332000, China; 20210204374@jju.edu.cn; 4 Department of Clinical Medicine, Grade 2023, School of Basic Medicine, Jiujiang University, Jiujiang 332000, China; 20230204439@jju.edu.cn

**Keywords:** DFUs, SIKVAV, chitosan hydrogels, skin wound healing, TGF-β1, Smad3

## Abstract

Diabetic foot ulcers (DFUs), a prevalent chronic disease caused by various factors, significantly impact patients’ quality of life due to prolonged healing times and increased infection risks. Current treatment modalities, including pharmacological, physical, and surgical interventions, often yield limited efficacy and adverse effects, highlighting the urgent need for novel therapeutic strategies. The objective of this research is to create SIKVAV-modified chitosan hydrogels with the intention of improving the process of skin wound healing in diabetic mice. We synthesized the hydrogels and established a diabetic mice model with skin wounding to evaluate its healing effects and underlying mechanisms. The results of our study indicate that the SIKVAV-modified chitosan hydrogels markedly enhance the wound healing process in diabetic mice. This effect may be attributed to several mechanisms, including differentiation of fibroblasts, proliferation of keratinocytes, the promotion of angiogenesis, stimulation of collagen synthesis, upregulation of growth factor expression, and possible involvement of the TGF-β1/Smad3 signaling pathway. This research not only provides a new biomaterial for the treatment of diabetic wounds but also elucidates the related molecular mechanisms involved in wound healing of DFUs, offering valuable insights for future clinical applications.

## 1. Introduction

Diabetes mellitus is a prevalent chronic disease that significantly impacts global health, with an increasing incidence rate worldwide. A significant complication linked to diabetes is the occurrence of DFUs, which can adversely affect the healing process of wounds and result in persistent ulcers and infections. The etiology of DFUs is complex, including wound infection, insufficient growth factors, limb ischemia, peripheral neuropathy, and other factors [1]. Research suggests that DFU patients endure extended healing durations, exhibiting greater vulnerability to infections, which ultimately impacts their quality of life and escalates healthcare expenses [2]. Current clinical treatments for DFUs primarily include pharmacological interventions, physical therapies, stem cell therapy, surgical options, and other methods [3]. However, these approaches often yield limited efficacy and may be accompanied by adverse effects, highlighting the urgent need for novel therapeutic strategies. The creation of novel materials that can improve the processes involved in wound healing is critically significant. Comprehending the fundamental processes of wound healing in individuals with diabetes could facilitate the development of more efficient therapies, consequently enhancing patient results and alleviating the strain of diabetic complications.

Chitosan, a naturally occurring polysaccharide obtained from chitin, has attracted considerable interest within the biomedical sector owing to its remarkable biocompatibility and biodegradability [4]. It has been extensively studied for its roles in wound healing, antibacterial properties, and hemostatic effects. The molecular mechanisms underlying chitosan’s efficacy in promoting wound healing are multifaceted, involving the enhancement of cell proliferation, migration, and angiogenesis [5]. A moist environment is more conducive to the healing of wounds. However, chitosan hydrogels can keep a wound moist, which promotes the proliferation of wound cells and angiogenesis. In diabetic wound healing, where the healing process is severely impaired, chitosan hydrogels have demonstrated potential in accelerating recovery by promoting tissue regeneration [6].

One of the protein components, laminin, comprises an α peptide chain containing the SIKVAV polypeptide sequence and two β peptides. Extensive experimental evidence has shown that SIKVAV has growth factor characteristics and plays a key role in the proliferation of various cells, including cell types such as vascular endothelial cells, fibroblasts, bone marrow-derived mesenchymal stem cells, and tumor cells, among others [7,8]. SIKVAV has the capacity to enhance the development of endothelial conduits within vascular endothelial cells in vitro, and this process is crucial for the process of angiogenesis. Many studies in vivo have shown that SIKVAV-modified biomaterials can promote the formation of blood vessels in skin wounds [9]. SIKVAV can promote the adhesion, neurite migration, and synaptic growth of neurons [10]. SIKVAV-modified 2-hydroxyethyl methacrylate hydrogels can promote the regeneration of spinal cord axons and promote the repair of nerve injury [11].

The TGF-β1/Smad3 signaling pathway is the most significant regulatory pathway in wound healing. Among several cytokines involved in wound healing, TGF-β1 plays the most extensive role and widely mediates various cellular functions [12]. During the process of wound healing, TGF-β1’s regulation varies depending on cells’ activation or differentiation state, affecting its role in chemotaxis, proliferation, adhesion, and other cellular activities. Furthermore, TGF-β1 exerts a bidirectional regulatory effect on the functions of target cells, thereby facilitating the wound healing process [13]. The most significant intracellular signal transduction protein of TGF-β1 is Smad3 protein. TGF-β1 has the capacity to directly stimulate the synthesis of extracellular matrix through a mechanism that is dependent on Smad3. Smad3 serves as a crucial protein involved in the regulation of cellular processes such as proliferation, differentiation, and apoptosis [14]. Furthermore, Smad3 protein promotes cell ultrastructure proliferation and differentiation, maintains intracellular stability, and accelerates wound tissue fibrosis. It also facilitates the growth of smooth muscle growth and enhances cell proliferation and migration, which is beneficial for the process of detumescence [15,16].

This study aims to develop SIKVAV-modified chitosan hydrogels and evaluate their effects and mechanisms in promoting skin wound healing in diabetic mice. Recent studies have highlighted the potential of SIKVAV-modified chitosan hydrogels in diabetic wound healing, demonstrating their ability to stimulate fibroblast differentiation and collagen synthesis. However, the specific molecular pathways through which SIKVAV-modified chitosan hydrogels exert their effects in diabetic conditions remain inadequately understood. Notably, the interaction of chitosan with various signaling pathways, such as TGF-β1 and its downstream Smad3 proteins, is an area that requires further exploration. Current research lacks comprehensive studies on mechanisms in diabetes, complicating healing. Understanding SIKVAV-modified chitosan hydrogels could lead to new DFU treatment strategies.

## 2. Results

### 2.1. SIKVAV-Modified Chitosan Hydrogels Significantly Improved the Healing Process of Skin Wounds in Diabetic Mice

**Figure 1:**
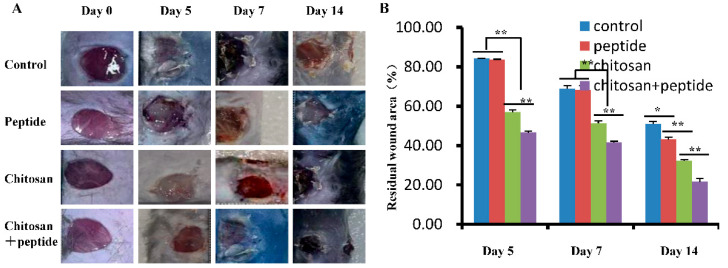
SIKVAV-modified chitosan hydrogels enhanced the contraction of skin wounds in diabetic mice. (**A**) Representative photographs of typical wounds among the control group, peptide group, chitosan group, and SIKVAV-modified chitosan group at 5, 7, and 14 days post-surgery; (**B**) statistical evaluation of the residual wound percentages in diabetic mice across the control group, peptide group, chitosan group, and SIKVAV-modified chitosan group (*n* = 3, * for *p* < 0.05, and ** for *p* < 0.01).

Figure 1A illustrates the contraction effect of SIKVAV-modified chitosan hydrogels on skin wounds in diabetic mice. Analysis of the alterations in wound dimensions indicates that the chitosan combined with a peptide group exhibited a superior wound healing effect compared to those in the other three experimental groups. The statistical analysis of the residual area of a skin wound in each group is shown in Figure 1B. In general, the area of the skin wounds in all groups exhibited a gradual reduction over time, with the chitosan combined with a peptide group showing a notably smaller residual wound area. These findings indicate that chitosan hydrogels modified with SIKVAV have the potential to enhance skin wound healing in diabetic mice.

**Figure 2:**
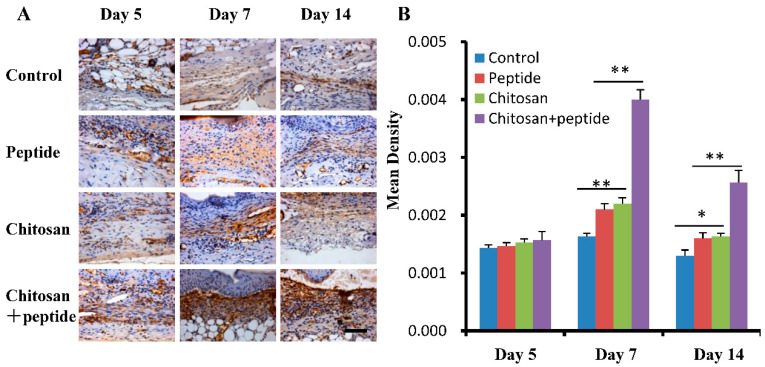
SIKVAV-modified chitosan hydrogels expedited the expression of α-SMA in the skin wounds of diabetic mice. (**A**) Immunohistochemistry illustrating the expression of α-SMA in the skin wounds across the control group, peptide group, chitosan group, and peptide-modified chitosan group at 5, 7, and 14 days post-surgery (scale bar: 50 μm); (**B**) statistical evaluation of α-SMA in the skin wounds among the control group, peptide group, chitosan group, and peptide-modified chitosan group at 5, 7, and 14 days following surgery (*n* = 3, * for *p* < 0.05, and ** for *p* < 0.01).

The process of skin wound contraction is significantly facilitated by the mechanical tension generated by myofibroblasts. Following traumatic injury, the wound healing cascade is triggered, leading to the development of granulation tissue. This process induces the differentiation of fibroblasts into contractile myofibroblasts, which play a crucial role in promoting wound contraction. Additionally, myofibroblasts exhibit marked expression of α-smooth muscle actin (α-SMA), further underscoring their importance in this physiological response. The chitosan + peptide group exhibited markedly elevated α-SMA expression compared to the other groups at 7 and 14 days post-trauma (Figure 2). The findings indicate that SIKVAV-modified chitosan hydrogels can enhance skin wound healing in diabetic mice, likely by stimulating α-SMA expression in wound tissues, thereby promoting wound contraction.

### 2.2. SIKVAV-Modified Chitosan Hydrogels Enhanced the Proliferation of Keratinocytes in the Skin Wounds of Diabetic Mice

**Figure 3:**
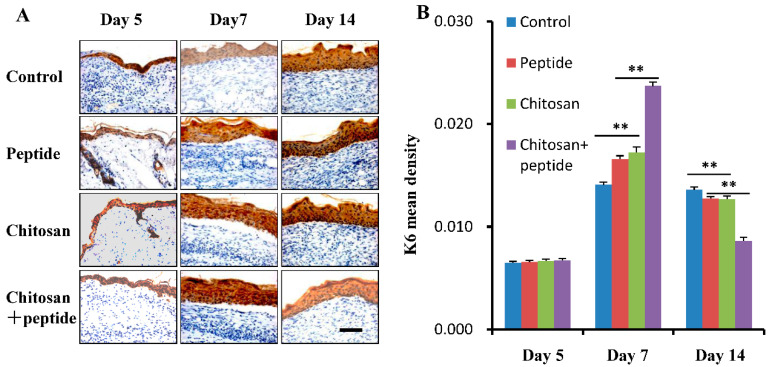
SIKVAV-modified chitosan hydrogels enhanced the proliferation of keratinocytes in the skin wounds of diabetic mice. (**A**) The levels of K6 expression in keratinocytes were evaluated in the control group, peptide group, chitosan group, and peptide-modified chitosan group using immunohistochemical techniques at 5, 7, and 14 days following surgery (scale bar: 50 μm). (**B**) The optical density of keratinocyte K6 was statistically assessed in the control group, the peptide group, the chitosan hydrogels group, and the peptide-modified chitosan hydrogels group at 5, 7, and 14 days post-surgery (*n* = 3, ** for *p* < 0.01).

K6 serves as an indicator of keratinocyte proliferation throughout the process of skin wound healing. To further validate the hypothesis that the SIKVAV-modified chitosan hydrogels promote keratinocyte proliferation in the skin wounds of diabetic mice, immunohistochemical methods were utilized to evaluate the expression levels of K6 in keratinocytes within the affected dermal regions of diabetic mice. The results are depicted in Figure 3A. Remarkably, the expression of K6 in keratinocytes across all groups was found to be reduced 5 days post-injury; however, a gradual increase was observed 7 days post-injury, followed by a subsequent decline in expression across all groups 14 days post-injury. Figure 3B illustrates quantitative analyses of the optical density of K6 in keratinocytes from the skin wounds across the four experimental groups at the intervals of 5, 7, and 14 days post-injury. At the five-day mark, no notable variations were observed in the optical density of K6 across the four experimental groups. In contrast, at both the seven- and fourteen-day intervals, the SIKVAV + chitosan group demonstrated a significantly higher optical density of K6 compared to the other groups. Additionally, no significant difference in the optical density of K6 was noted between the SIKVAV and chitosan groups at the five-day post-injury time point.

### 2.3. SIKVAV-Modified Chitosan Hydrogels Enhanced Angiogenesis and Collagen Production in the Skin Wounds of Diabetic Mice

**Figure 4:**
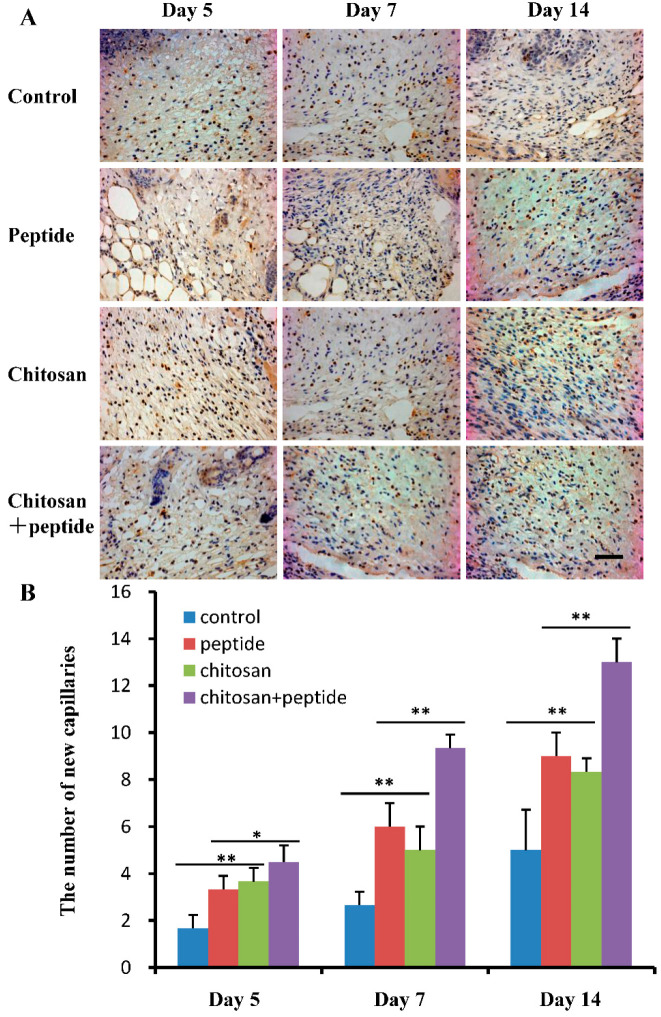
SIKVAV-modified chitosan hydrogels enhanced angiogenesis in the skin wounds of diabetic mice. (**A**) Immunohistochemical evaluation of CD31 expression in vascular endothelial cells of the skin wounds at 5, 7, and 14 days post-surgery among the control group, the peptide group, the chitosan group, and the SIKVAV-modified chitosan group (scale bar: 50 μm); (**B**) statistical assessment of new blood capillaries among the control group, peptide group, chitosan group, and SIKVAV-modified chitosan group (*n* = 3, * for *p* < 0.05, and ** for *p* < 0.01).

The development of new blood vessels in skin wounds is essential for delivering nutrients required for the formation of granulation tissue and the proliferation of keratinocytes, both of which are essential components in the process of skin wound healing. In the current study, immunohistochemical methods were utilized to detect the expression of CD31 within the endothelial cells of capillaries found in skin wounds, thereby enabling examination of angiogenesis. The findings are illustrated in Figure 4A. Notably, the angiogenic response observed in the SIKVAV + chitosan group exceeded those of the control, SIKVAV, and chitosan groups. A statistical analysis detailing the number of newly formed blood capillaries in the skin wounds of diabetic mice is illustrated in Figure 4B. Specifically, the SIKVAV + chitosan group demonstrated a significantly higher count of new blood capillaries in the skin wounds at 5, 7, and 14 days post-injury compared to the control, SIKVAV, and chitosan groups, while no notable difference was detected between the SIKVAV and chitosan groups. These results strongly indicate that the SIKVAV-modified chitosan hydrogels significantly enhance angiogenesis in the skin wounds of diabetic mice. 

**Figure 5:**
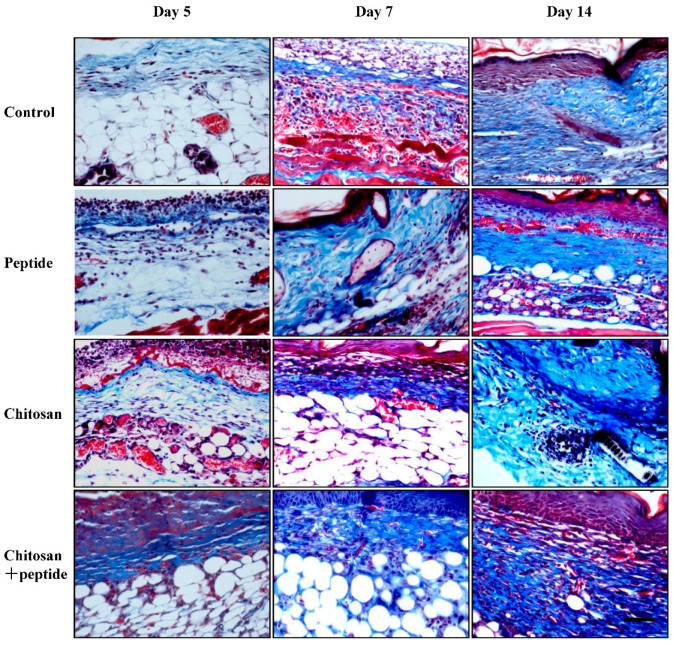
Masson trichrome staining demonstrated the presence of newly synthesized collagen at 5, 7, and 14 days post-treatment in the skin wounds of diabetic mice across the control group, the peptide group, the chitosan group, and the SIKVAV-modified chitosan group (scale bar: 50 μm).

The synthesis of collagen is crucial for efficient skin wound healing, as it provides a structural framework that facilitates cellular proliferation and the regeneration of blood vessels. In the present investigation, Masson’s trichrome staining was employed to observe the development of new collagen fibers in the skin wounds of diabetic mice. As illustrated in Figure 5, on days 5, 7, and 14 following the injury, the granulation tissue of the skin wounds in the SIKVAV + chitosan group exhibited a higher density of collagen fibers when compared to that in the other three experimental groups. Significantly, on day 7 following the injury, there was a marked increase in collagen fiber deposition in the SIKVAV + chitosan group, while the control, SIKVAV, and chitosan groups exhibited a diminished presence of collagen fibers. By the fourteenth day following the injury, a significant presence of collagen fibers was observed in the skin wounds of the group treated with SIKVAV + chitosan. These findings suggest that the SIKVAV-modified chitosan hydrogels substantially promote the deposition of collagen fibers in the skin wounds of diabetic mice.

### 2.4. SIKVAV-Modified Chitosan Hydrogels Enhanced the Expression of Wound Growth Factor mRNA in Diabetic Mice

**Figure 6:**
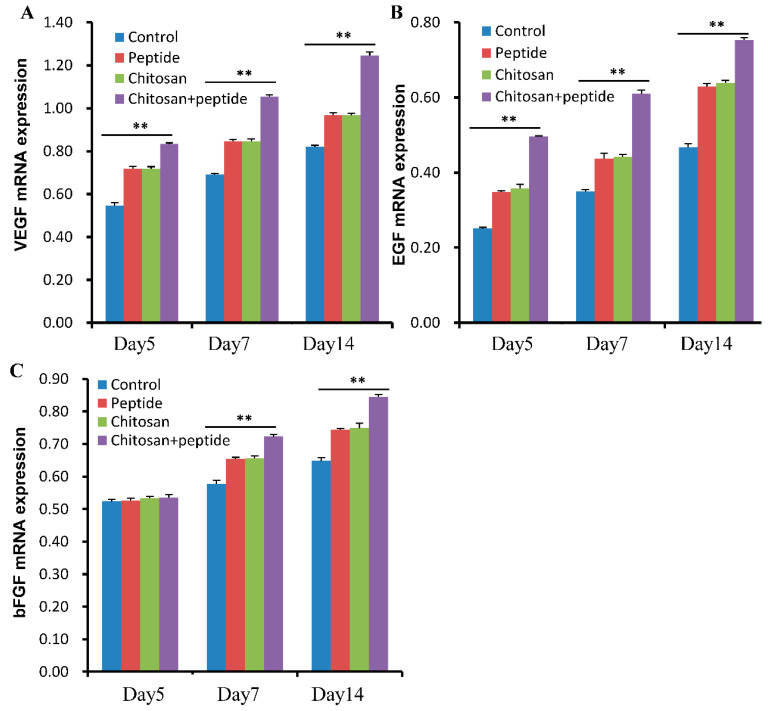
SIKVAV-modified chitosan hydrogels significantly increased the mRNA expression levels of growth factors in the skin wounds of diabetic mice at 5, 7, and 14 days post-treatment. This enhancement includes the mRNA levels of (**A**) VEGF, (**B**) bFGF, and (**C**) EGF (*n* = 3, ** for *p* < 0.01).

The process of skin wound healing involves a diverse array of growth factors and related proteins, including vascular endothelial growth factor (VEGF), epidermal growth factor (EGF), and basic fibroblast growth factor (bFGF). During the skin wound repair process, these agents can facilitate wound angiogenesis, keratinocyte proliferation, collagen synthesis, and more. The mRNA levels of growth factors such as VEGF, EGF, and bFGF demonstrated an increasing pattern over the observed time period, with the expression of these factors in the chitosan + SIKVAV group exceeding that in the other experimental groups (Figure 6). This finding indicates that SIKVAV-modified chitosan hydrogels can enhance the mRNA expression of growth factors VEGF, EGF, and bFGF in the skin wounds of diabetic mice.

### 2.5. SIKVAV-Modified Chitosan Hydrogels Facilitated Skin Wound Healing in Diabetic Mice via the TGF-β1/Smad3 Signaling Pathway

**Figure 7:**
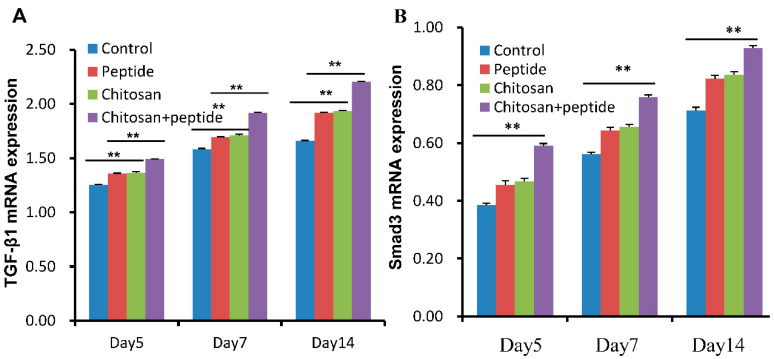
SIKVAV-modified chitosan hydrogels increased the expression levels of TGF-β1 (**A**) and Smad3 (**B**) mRNA in the skin wounds of diabetic mice at 5, 7, and 14 days post-initial treatment (*n* = 3, ** for *p* < 0.01).

**Figure 8:**
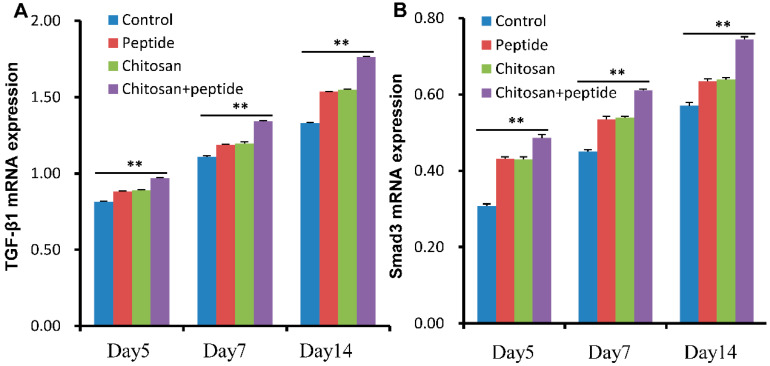
After subcutaneous injection of lentivirus that interferes with the expression of TGF-β1, SIKVAV-modified chitosan hydrogels accelerated the expression levels of TGF-β1 (**A**) and Smad3 (**B**) mRNA in the skin wounds of diabetic mice at 5, 7, and 14 days following initial treatment (*n* = 3, ** for *p* < 0.01).

TGF-β1 and Smad3 proteins are involved in angiogenesis, collagen synthesis, and keratinocyte proliferation in the process of skin wound repair. Based on the abovementioned results, the SIKVAV-modified chitosan hydrogels can promote the mRNA expression of both TGF-β1 and Smad3 in wounds (Figure 7). In the present investigation, a lentiviral vector was administered via subcutaneous injection to modulate the mRNA expression levels of TGF-β1. This approach aimed to assess the mRNA expression of both TGF-β1 and Smad3 within the skin wounds of diabetic murine models. The result shown that the chitosan + peptide group had significantly higher TGF-β1 and Smad3 mRNA expression than the other three groups (Figure 8). The findings indicate that chitosan hydrogels modified with SIKVAV facilitate the healing of skin wounds in diabetic mice via the TGF-β1/Smad3 signaling pathway.

## 3. Discussion

Individuals diagnosed with diabetes are more likely to develop DFUs. DFUs form through a multifactorial and multi-link process involving the epidermis, subcutaneous tissue, extracellular matrix, multiple cytokines, infection, peripheral vascular disease, peripheral neuropathy, etc. [1]. Due to the challenges associated with treatment and the frequent lack of successful healing, unfortunately, approximately 25% of patients with DFUs are at risk of amputation. This condition is the main cause of disability among individuals with diabetes, significantly impairing their quality of life [17,18]. The healing difficulties associated with DFUs represent a significant clinical challenge, as patients often experience prolonged recovery times and increased susceptibility to infections [19]. Current treatment modalities, including pharmacological, physical, and surgical interventions, among others [3], have shown limited efficacy and are often accompanied by adverse effects. Consequently, there is a pressing need for innovative therapeutic strategies that can effectively enhance wound healing in diabetic patients. 

TGF-β1 is recognized for its role in promoting the proliferation and differentiation of fibroblasts, as well as enhancing collagen synthesis [20,21]. The findings of this research show that the SIKVAV-modified chitosan hydrogels significantly accelerate the healing process (Figure 1), fibroblast differentiation (Figure 2), keratinocyte proliferation (Figure 3), and collagen synthesis (Figure 5) in the skin wounds of diabetic mice. The findings of this study indicate that the observed increase in TGF-β1 and its downstream mediator Smad3 implies that the SIKVAV-modified chitosan hydrogels promote progression into the proliferative phase of the wound healing process. This is particularly important in DFUs, where the healing process is often impaired due to chronic inflammation and reduced cellular responsiveness. This improvement in wound closure indicates that the hydrogels hold significant promise for clinical use as an innovative treatment alternative for diabetic wounds. 

In patients with diabetes, the efficacy of monotherapy is frequently limited due to the interplay of various pathological mechanisms [22]. Suboptimal healing responses are often observed, as traditional treatments may not adequately address the underlying complications. As a result, DFUs represent a clinical challenge involving a complex regulatory network. It is important to emphasize that TGF-β1 plays a crucial role in DFUs. This cytokine facilitates angiogenesis, thereby impacting the process of wound healing [12,23]. Dysregulation in this pathway can impair neovascularization, further exacerbating ulceration and delaying tissue repair. Additionally, the activation of the TGF-β1/Smad3 pathway may also contribute to angiogenesis, as TGF-β1 is implicated in the formation of new blood vessels [24]. A significant contributor to DFUs is the presence of peripheral microvascular damage. The SIKVAV-modified chitosan hydrogels continuously release SIKVAV during wound healing, which promotes angiogenesis in skin wounds [9]. The observed increase in CD31 expression, a marker for endothelial cells, further supports the notion that the SIKVAV-modified chitosan hydrogels enhance vascularization in the wound area (Figure 4). Despite the advancements in understanding DFUs, the relationship between ulcers and vascular regeneration mechanisms remains a critical focus for ongoing research. Deepened understanding of the interaction between vascular growth and wound healing can facilitate the development of groundbreaking therapeutic approaches, ultimately enhancing results for patients afflicted by diabetic foot ulcers. 

DFUs present a complex clinical challenge characterized by impaired wound healing, where multiple growth factors play critical roles [25]. Among these, TGF-β1 is a pivotal mediator in the healing process, closely interacting with various growth factors such as VEGF, bFGF, and EGF. Research has indicated that TGF-β1 can enhance the production of these growth factors [12,23,26]. Wound repair involves the above growth factors. These cytokines can promote wound angiogenesis, fibroblast synthesis, collagen fiber deposition, and keratinocyte proliferation. These growth factors promote wound repair through multiple pathways. However, the presence of these growth factors alone is insufficient for DFU wounds. Our findings demonstrate that the SIKVAV-modified chitosan hydrogels can enhance the expression of these factors within the skin wounds of diabetic mice (Figure 6 and Figure 7A). 

The TGF-β1/Smad3 signaling pathway plays a crucial role in the healing process of DFUs [27]. TGF-β1 serves not only to stimulate these healing processes but also to modulate the expression of angiogenic factors. It has been found that TGF-β1 can enhance VEGF expression, facilitating neovascularization and thereby improving oxygenation to healing tissue [23]. In parallel, bFGF and EGF, both of which promote endothelial and keratinocyte proliferation, are influenced by TGF-β1 [26]. This relationship emphasizes the interconnected roles of these factors in establishing a conducive environment for healing. In order to further observe the role of TGF-β 1/Smad3 in the repair of skin wounds in diabetic mice, we manipulated the expression levels of TGF-β1 through subcutaneous administration of a lentivirus. The findings indicate that the SIKVAV-modified chitosan hydrogels significantly enhanced the expression of both TGF-β1 and Smad3 mRNA (Figure 8) in comparison to that in the other experimental groups. The results suggest that the SIKVAV-modified chitosan hydrogels significantly enhance wound healing in diabetic mice, which may be related to the TGF-β1/Smad3 signaling pathway. This signaling pathway is essential for numerous cellular activities, such as cell growth, differentiation, the formation of new blood vessels, and the synthesis of the extracellular matrix, all of which are critical for successful wound healing. 

The constraints of this study predominantly arise from its dependence on a murine model, highlighting the need for additional validation through more extensive animal studies and clinical trials to ascertain the effectiveness of the SIKVAV-modified chitosan hydrogels. Additionally, while the focus was placed on the TGF-β1/Smad3 signaling pathway, skin wound repair is a complex process influenced by various factors, such as the cutaneous stress response system and neuropeptides involved in skin neuroendocrine signaling [28]. Therefore, further research is needed to explore other potential molecular mechanisms and comprehensively elucidate the therapeutic advantages of the SIKVAV-modified chitosan hydrogels in DFUs. 

## 4. Materials and Methods

### 4.1. Materials

Chitosan, with an 85% deacetylation degree and a molecular weight of 100,000 Da, was sourced from Golden Shell Pharmaceutical Co., Ltd. located in Yuhuan, China. Methacrylic anhydride was acquired from APC Chemicals Company based in Montreal, Canada. The compound 3-(Maleimido) propionic acid *n*-hydroxysuccinimide ester (SMP; 97%) was obtained from Polysciences Corporation in Tamil Nadu, India. Additionally, *N*,*N*,*N*,*N*-tetramethyl ethylenediamine (TEMED), ammonium persulfate (APS), and dimethylformamide (DMF) were procured from Sigma Aldrich in Guangzhou, China. The peptide SIKVAV was supplied by Shanghai Kopeptide Biotechnology Co., Ltd. (China, Shanghai). The α-SMA antibody was obtained from Wuhan Bode Reagent Co., Ltd. (China, Wuhan), while the CD31 monoclonal antibody was sourced from Dako in Guangzhou, China. Finally, the K6 polyclonal antibody was purchased from Convance, based in New York, NY, USA. The secondary antibodies corresponding to primary antibodies, including CD31, K6, and α-SMA, and an immunohistochemical kit were purchased from Wuhan Bode Reagent Co., Ltd.; TRIzol reagent and the cDNA reverse transcription kit were acquired from Invitrogen, located in Carlsbad, CA, USA. Monoclonal antibodies specific to TGF-β1 and Smad3 were sourced from Cell Signaling Technology (CST), (Boston, MA, USA). Additionally, the cDNA Synthesis Kit and SYBR Green were obtained from Roche, Switzerland. Quantitative reverse transcription polymerase chain reaction (qRT-PCR) test kits for the detection of EGF mRNA, bFGF mRNA, TGF-β1 mRNA, Smad3 mRNA, VEGF mRNA, and GAPDH were procured from Shanghai Lichen Biotechnology Company based in Shanghai, China. The lentivirus utilized in this study was acquired from Wuhan Bode Reagent Co., Ltd.

### 4.2. Preparation of Peptide SIKVAV-Modified Chitosan Hydrogels

The preparation of the SIKVAV-modified chitosan hydrogels was conducted following the protocols outlined in our earlier publications [9,29]. In summary, first, 50 mg/mL of peptide SIKVAV-modified chitosan solution and 100 mg/mL of ammonium persulfate solution were prepared to a final volume of 1 mL each. Subsequently, 440 μL of 50 mg/mL double-bonded chitosan aqueous solution was added into a 1.5 mL centrifuge tube and blown evenly using a pipette. Subsequently, 4.5 μL of ammonium persulfate was introduced and uniformly dispersed using a pipette. Finally, 0.5 μL of TEMED solution was added, oscillated using an oscillator for 10 s, and placed for 30 min to form hydrogels. Please refer to the Supplementary Materials.

### 4.3. Establishment of a Skin Injury Model in Diabetic Mice 

Animal experiments were conducted at the Animal Experimental Center of Jiujiang University, with approval obtained from the Medical Ethics Committee of the same institution. This procedure adhered strictly to the NIH guidelines concerning the management and safety of laboratory animals. The db/db mice with diabetes utilized in this investigation were sourced from the Model Animal Research Institute at Nanjing University. A wound model with a diameter of six millimeters was created on the dorsal region of the mice. After the trauma model was successfully established, the mice were randomly divided into four distinct groups: the control group, which received no treatment for the wound; the peptide group, where the SIKVAV solution was solely applied to the wound; and the chitosan group in which the wound was dressed with chitosan hydrogels; and chitosan + peptide group, where the wound was dressed with SIKVAV-modified chitosan hydrogels. The intervention for the TGF-β1 expression experiment was carried out via subcutaneous injection of a lentivirus, and the remainder of the experimental process was as previously described. Each mouse was kept in solitary confinement and was provided with unlimited access to both food and water. The wound site was captured through a digital camera on the 5th, 7th, and 14th days post-trauma, and the wound area proportion was computed alongside the remaining skin wound area ratio utilizing Equation (1).

(%) = St/So × 100%(1)

The ratio of the remaining area of a wound. So: the initial size of the injury, St: the residual surface area of the wound at various time intervals.

### 4.4. Immunohistochemical Staining

A five-micrometer paraffin section was dewaxed with xylene, rehydrated, and then subjected to antigen retrieval. Then, 10% H_2_O_2_ inactivated the endogenous enzyme for 10 min, and 5% BSA was applied for 2 h. The primary antibodies, specifically CD31, K6, and α-SMA, were incubated overnight at 4 °C and subsequently washed with PBS. The corresponding secondary antibodies were selected on the basis of the primary antibodies, which were subsequently incubated for 2 h and cleaned with PBS. The SABC reaction was conducted for 20 min, followed by DAB staining. The sections were then counterstained with hematoxylin, dehydrated with gradient alcohol, cleared with xylene, and embedded in resin. Finally, the samples were examined under a microscope. 

### 4.5. Masson Trichromatic Staining

The paraffin section underwent a standard procedure of dewaxing to remove paraffin and was subsequently rehydrated in water. It was stained with hematoxylin for a duration of 15 min, followed by thorough washing. Masson blue solution was then applied, restoring the blue color, after which another washing step was performed. Following a brief period of color separation using 1% hydrochloric acid alcohol, the section was gently rinsed with double-distilled water (ddH_2_O). The staining process continued with Ponceau for 5 min, followed by extensive rinsing. The specimen was then immersed in a 1% phosphomolybdic acid solution for 5 min and agitated until dry. Finally, it was stained with aniline blue for an additional 5 min before being washed with water. Subsequently, 95% ethanol was used for rapid dehydration, and then anhydrous ethanol was applied for dehydration for 10 s three times. Once the xylene achieved transparency, the film was subsequently encapsulated using a neutral resin.

### 4.6. Real-Time Fluorescence Quantitative PCR

The gene expression of growth factors including EGF mRNA, bFGF mRNA, VEGF mRNA, TGF-β1 mRNA, and Smad3 mRNA in the mouse wound tissues were detected using qRT-PCR. From each group, 100 mg of wound tissues was taken and ground into powder using liquid nitrogen, 1 mL of TRIzol was added, and RNA was extracted. In every group, 1 μg of RNA underwent reverse transcription to synthesize cDNA. The assessment of gene expression levels was performed in accordance with the specified experimental protocols. 

### 4.7. Statistical Methods

Statistical analyses were conducted utilizing SPSS version 20.0 (International Business Machines Corporation, Guangzhou, China). Measurement data were presented as the means accompanied by standard deviations. An independent sample *t*-test was employed to compare the means between two samples. For the comparison of means across multiple samples, one-way analysis of variance was utilized. Post-hoc comparisons among multiple groups were performed using the Least Significant Difference (LSD) method when variances were homogeneous, and Tamhane’s method was applied in cases of variance heterogeneity. *p* < 0.05 was considered statistically significant. All experiments were performed at least three times.

## 5. Conclusions

The etiology of DFUs is complex, and the lack of growth factors plays an important role. The results indicate that SIKVAV-modified chitosan hydrogels exhibit notable effectiveness in promoting wound healing in diabetic murine models. This bioengineered material accelerates dermal recovery by promoting fibroblast differentiation, collagen synthesis, keratinocyte proliferation, angiogenesis, and growth factor secretion and potentially participating in the TGF-β1/Smad3 signaling pathway. This research not only enhances the comprehension of the molecular mechanisms involved in the wound healing process but also underscores the potential therapeutic benefits and clinical applications of SIKVAV-modified biomaterials in managing complications related to DFUs. By clarifying the advantageous impacts of the SIKVAV-modified chitosan hydrogels, this study lays the groundwork for forthcoming explorations focused on enhancing wound treatment approaches for DFU management.

## Data Availability

Data are contained within the article and Supplementary Materials.

## Supplementary Materials

The following supporting information can be downloaded at: https://www.mdpi.com/article/10.3390/molecules29225374/s1.

## References

[1] Deng H., Li B., Shen Q., Zhang C., Kuang L., Chen R., Wang S., Ma Z., Li G. (2023). Mechanisms of diabetic foot ulceration: A review. J. Diabetes.

[2] Wang X., Yuan C.X., Xu B., Yu Z. (2022). Diabetic foot ulcers: Classification, risk factors and management. World J. Diabetes.

[3] Yang L., Rong G.C., Wu Q.N. (2022). Diabetic foot ulcer: Challenges and future. World J. Diabetes.

[4] Tang W., Wang J., Hou H., Li Y., Wang J., Fu J., Lu L., Gao D., Liu Z., Zhao F. (2023). Review: Application of chitosan and its derivatives in medical materials. Int. J. Biol. Macromol..

[5] Guo S., Ren Y., Chang R., He Y., Zhang D., Guan F., Yao M. (2022). Injectable Self-Healing Adhesive Chitosan Hydrogel with Antioxidative, Antibacterial, and Hemostatic Activities for Rapid Hemostasis and Skin Wound Healing. ACS Appl. Mater. Interfaces.

[6] Wei X., Liu C., Li Z., Gu Z., Yang J., Luo K. (2024). Chitosan-based hydrogel dressings for diabetic wound healing via promoting M2 macrophage-polarization. Carbohydr. Polym..

[7] Boccafoschi F., Fusaro L., Mosca C., Bosetti M., Chevallier P., Mantovani D., Cannas M. (2012). The biological response of poly(L-lactide) films modified by different biomolecules: Role of the coating strategy. J. Biomed. Mater. Res. Part A.

[8] Kubinova S., Horak D., Vanecek V., Plichta Z., Proks V., Sykova E. (2014). The use of new surface-modified poly(2-hydroxyethyl methacrylate) hydrogels in tissue engineering: Treatment of the surface with fibronectin subunits versus Ac-CGGASIKVAVS-OH, cysteine, and 2-mercaptoethanol modification. J. Biomed. Mater. Res. Part A.

[9] Chen S., Zhang M., Shao X., Wang X., Zhang L., Xu P., Zhong W., Zhang L., Xing M., Zhang L. (2015). A laminin mimetic peptide SIKVAV-conjugated chitosan hydrogel promoting wound healing by enhancing angiogenesis, re-epithelialization and collagen deposition. J. Mater. Chem. B.

[10] He L., Liao S., Quan D., Ngiam M., Chan C.K., Ramakrishna S., Lu J. (2009). The influence of laminin-derived peptides conjugated to Lys-capped PLLA on neonatal mouse cerebellum C17.2 stem cells. Biomaterials.

[11] Kubinova S., Horak D., Hejcl A., Plichta Z., Kotek J., Proks V., Forostyak S., Sykova E. (2015). SIKVAV-modified highly superporous PHEMA scaffolds with oriented pores for spinal cord injury repair. J. Tissue Eng. Regen. Med..

[12] Li Y., Duan W., Shen L., Ma X., Ma J., Zhang Y., Guo Y. (2023). Shengji solution accelerates the wound angiogenesis of full-thickness skin defect in rats via activation of TGF-beta1/Smad3-VEGF signaling pathway. Biotechnol. Genet. Eng. Rev..

[13] Song S., Shi C., Bian Y., Yang Z., Mu L., Wu H., Duan H., Shi Y. (2022). Sestrin2 remedies podocyte injury via orchestrating TSP-1/TGF-beta1/Smad3 axis in diabetic kidney disease. Cell Death Dis..

[14] Chen J., Xia Y., Lin X., Feng X.H., Wang Y. (2014). Smad3 signaling activates bone marrow-derived fibroblasts in renal fibrosis. Lab. Investig. A J. Tech. Methods Pathol..

[15] Liu H., Yong Y., Li X., Ye P., Tao K., Peng G., Mo M., Guo W., Chen X., Luo Y. (2022). Chaperone-mediated Autophagy Regulates Cell Growth by Targeting SMAD3 in Glioma. Neurosci. Bull..

[16] Xu B.H., Sheng J., You Y.K., Huang X.R., Ma R.C.W., Wang Q., Lan H.Y. (2020). Deletion of Smad3 prevents renal fibrosis and inflammation in type 2 diabetic nephropathy. Metab. Clin. Exp..

[17] Bandyk D.F. (2018). The diabetic foot: Pathophysiology, evaluation, and treatment. Semin. Vasc. Surg..

[18] Ajmeer A.S., Dudhamal T.S., Gupta S.K. (2015). Management of Madhumehajanya Vrana (diabetic wound) with Katupila (Securinega leucopyrus [Willd] Muell.) Kalka. Ayu.

[19] Da Ros R., Volpe A., Bordieri C., Tramonta R., Bernetti A., Scatena A., Monge L., Ragghianti B., Silverii A., Uccioli L. (2024). Prevention of foot ulcers recurrence in patients with diabetes: A systematic review and meta-analysis of randomized controlled trials for the development of the italian guidelines for the treatment of diabetic foot syndrome. Acta Diabetol..

[20] Fu X., Xu M., Jia C., Xie W., Wang L., Kong D., Wang H. (2016). Differential regulation of skin fibroblasts for their TGF-β1-dependent wound healing activities by biomimetic nanofibers. J. Mater. Chem. B.

[21] Song Z., Yu T., Ge C., Shen X., Li P., Wu J., Tang C., Liu T., Zhang D., Li S. (2023). Advantage effect of Dalbergia pinnata on wound healing and scar formation of burns. J. Ethnopharmacol..

[22] Folli F., Finzi G., Manfrini R., Galli A., Casiraghi F., Centofanti L., Berra C., Fiorina P., Davalli A., La Rosa S. (2023). Mechanisms of action of incretin receptor based dual- and tri-agonists in pancreatic islets. Am. J. Physiol. Endocrinol. Metab..

[23] Chang M., Nguyen T.T. (2021). Strategy for Treatment of Infected Diabetic Foot Ulcers. Acc. Chem. Res..

[24] Zhao Q., Xu J., Han X., Zhang Z., Qu J., Cheng Z. (2022). Growth differentiation factor 10 induces angiogenesis to promote wound healing in rats with diabetic foot ulcers by activating TGF-beta1/Smad3 signaling pathway. Front. Endocrinol. (Lausanne).

[25] Li T., Ma Y., Wang M., Wang T., Wei J., Ren R., He M., Wang G., Boey J., Armstrong D.G. (2019). Platelet-rich plasma plays an antibacterial, anti-inflammatory and cell proliferation-promoting role in an in vitro model for diabetic infected wounds. Infect. Drug Resist..

[26] Cecerska-Heryc E., Goszka M., Serwin N., Roszak M., Grygorcewicz B., Heryc R., Dolegowska B. (2022). Applications of the regenerative capacity of platelets in modern medicine. Cytokine Growth Factor Rev..

[27] Viana-Mendieta P., Sanchez M.L., Benavides J. (2022). Rational selection of bioactive principles for wound healing applications: Growth factors and antioxidants. Int. Wound J..

[28] Slominski A.T., Slominski R.M., Raman C., Chen J.Y., Athar M., Elmets C. (2022). Neuroendocrine signaling in the skin with a special focus on the epidermal neuropeptides. Am. J Physiol Cell Physiol.

[29] Chen X., Cao X., Jiang H., Che X., Xu X., Ma B., Zhang J., Huang T. (2018). SIKVAV-Modified Chitosan Hydrogel as a Skin Substitutes for Wound Closure in Mice. Molecules.

