# Supplementary material for: Enhancing Skin Wound Healing in Diabetic Mice Using SIKVAV-Modified Chitosan Hydrogels
Source: Molecules. 2024 Nov 14;29(22):5374. doi: 10.3390/molecules29225374 (PMC11596890; doi:10.3390/molecules29225374)
Supplement: Supplementary file 1 [file molecules-29-05374-s001.zip › molecules-3243370-supplementary.pdf]

Supplementary Information

1. Synthesis of the first double bond chitosan

Dissolve 3g of chitosan in 100mL of 3% glacial acetic acid solution, heat to 50 ℃ and stir for 3 hours. After the chitosan dissolves, cool at room temperature. Then add 1.1mL of methacrylic anhydride and stir at room temperature for 3 hours. Then add the obtained mixture to a dialysis bag with a molecular weight of 8000-12000 for dialysis for 2 days, changing the water three times a day. Finally, freeze dry the dialysis solution and store the obtained sample at -20 ℃ for future use.

2) Preparation of the second chitosan hydrogels.

Prepare 1mL of 50 mg/mL double bond chitosan aqueous solution and 1mL of 100 mg/mL ammonium persulfate solution. The preparation method of double bond chitosan hydrogels is as follows: add 440 uL 50 mg/mL double bond chitosan aqueous solution into a 1.5 mL centrifuge tube, and blow it evenly with a pipette gun. Then add 4.5 uL of ammonium persulfate and blow evenly with a pipette. Finally, add 0.5 uL of TEMED solution, shake with an oscillator for 10 seconds, and let it stand for 30 minutes. Prepare 50 mg/mL double bond chitosan hydrogel.

3) The synthesis of double bond chitosan modified with a third maleimide group.

Firstly, dissolve 100 mg of double bond chitosan in 10 mL of distilled water. Dissolve 15 mg of 3-maleimidopropionic acid-N-succinimide ester in 500 uL of N-dimethylformamide (DMF) and add it to the double bond chitosan aqueous solution in 5 portions, with an interval of 1 hour between each addition. Stir overnight at room temperature. Finally, dialyze the obtained liquid and freeze dry it, then store it at -20 ℃ for future use.

4) Synthesis of chitosan modified with fourth peptide SIKVAV.

Dissolve 100 mg of double bond chitosan modified with maleimide groups in 10 mL of anaerobic water, then add 20 mg of peptide SIKVAV (the ratio of chitosan to peptide SIKVAV is calculated as 5:1 based on the substitution rate of chitosan double bonds and the binding rate between SMP and peptide SIKVAV), and stir at room temperature for 24 hours under nitrogen protection. Finally, dialyze the obtained liquid and freeze dry it, then store it at -20 ℃.

5) The fifth polypeptide SIKVAV modified chitosan hydrogels was prepared.

Firstly, prepare 1ml each of a 50 mg/mL peptide SIKVAV modified chitosan solution and a 100 mg/mL ammonium persulfate solution. Add 440 uL of 50 mg/mL double bond chitosan aqueous solution into a 1.5 mL centrifuge tube and blow evenly with a pipette. Then add 4.5 uL of ammonium persulfate and blow evenly with a pipette. Finally, 0.5 uL TEMED solution was added, shaken with an oscillator for 10s, and left for 30min to form hydrogels.
